# High-flux hemodialysis with polymethylmethacrylate membranes reduces soluble CD40L, a mediator of cardiovascular disease in uremia

**DOI:** 10.1093/ndt/gfaf101

**Published:** 2025-06-06

**Authors:** Marita Marengo, Massimiliano Migliori, Guido Merlotti, Erika Naso, Sergio Dellepiane, Davide Medica, Giuseppe Cappellano, Simone Cortazzi, Andrea Colombatto, Alessandro D Quercia, Colombano Sacco, Gianluca Leonardi, Olga Randone, Stefano Maffei, Elvira Mancini, Maurizio Borzumati, Paolo Fabbrini, Matteo Vidali, Elena Grossini, Claudio Medana, Federica Dal Bello, Marco Quaglia, Vincenzo Panichi, Vincenzo Cantaluppi

**Affiliations:** Nephrology and Dialysis Unit, ASL CN1, Cuneo, Italy; Nephrology and Dialysis Unit, Versilia Hospital ASL Toscana Nord-Ovest, Camaiore (LU), Italy; Department of Primary Care, ASST Pavia, Pavia, Italy; Nephrology and Dialysis Unit, ASL CN1, Cuneo, Italy; Nephrology and Kidney Transplantation Unit, Department of Translational Medicine, University of Piemonte Orientale (UPO), “Maggiore della Carità” University Hospital, Novara, Italy; Nephrology and Kidney Transplantation Unit, Department of Translational Medicine, University of Piemonte Orientale (UPO), “Maggiore della Carità” University Hospital, Novara, Italy; Nephrology and Kidney Transplantation Unit, Department of Translational Medicine, University of Piemonte Orientale (UPO), “Maggiore della Carità” University Hospital, Novara, Italy; Nephrology and Kidney Transplantation Unit, Department of Translational Medicine, University of Piemonte Orientale (UPO), “Maggiore della Carità” University Hospital, Novara, Italy; Nephrology and Kidney Transplantation Unit, Department of Translational Medicine, University of Piemonte Orientale (UPO), “Maggiore della Carità” University Hospital, Novara, Italy; Nephrology and Dialysis Unit, ASL CN1, Cuneo, Italy; Nephrology and Dialysis Unit, ASL BI, Biella, Italy; Nephrology and Dialysis Unit, ASL TO5, Chieri (TO), Italy; Nephrology and Dialysis Unit, ASL AT, Asti, Italy; Nephrology and Dialysis Unit, ASL AT, Asti, Italy; Nephrology and Dialysis Unit, ASL VCO, Verbania, Italy; Nephrology and Dialysis Unit, ASL VCO, Verbania, Italy; Nephrology and Dialysis Unit, Bassini Hospital, Cinisello Balsamo (MI), Italy; Nephrology and Kidney Transplantation Unit, Department of Translational Medicine, University of Piemonte Orientale (UPO), “Maggiore della Carità” University Hospital, Novara, Italy; Nephrology and Kidney Transplantation Unit, Department of Translational Medicine, University of Piemonte Orientale (UPO), “Maggiore della Carità” University Hospital, Novara, Italy; Unit of Mass Spectrometry, Department of Molecular Biotechnology and Health Sciences, University of Torino, Italy; Unit of Mass Spectrometry, Department of Molecular Biotechnology and Health Sciences, University of Torino, Italy; Nephrology and Dialysis Unit, “SS Biagio e Antonio e Cesare Arrigo” University Hospital, University of Piemonte Orientale (UPO), Alessandria, Italy; Nephrology, Dialysis and Transplantation Unit, Department of Clinical and Experimental Medicine, University of Pisa, Italy; Nephrology and Kidney Transplantation Unit, Department of Translational Medicine, University of Piemonte Orientale (UPO), “Maggiore della Carità” University Hospital, Novara, Italy

**Keywords:** hemodialysis, major adverse cardiovascular events (MACE), polymethylmethacrylate (PMMA) membrane, soluble CD40 ligand, vascular aging

## Abstract

**Background and hypothesis:**

Major adverse cardiovascular events (MACE) are the main cause of mortality in hemodialysis (HD). Soluble CD40 ligand (sCD40L) binds to CD40 on endothelial cells (EC) and vascular smooth muscle cells (VSMC), playing a potential role in MACE. HD registries show a reduced mortality for MACE using the polymethylmethacrylate (PMMA) membrane. Study objectives were (i) to confirm the role of sCD40L as independent predictor and mediator of MACE and (ii) to evaluate the effect of PMMA on sCD40L-mediated vascular aging.

**Methods:**

In 201 patients treated by high-flux HD, sCD40L levels were measured and correlated with MACE; 54/201 patients with sCD40L greater than or equal to the median value were randomized for 9 months in two crossover groups alternatively treated with PMMA or polysulfone (PS): sCD40L and dialytic parameters were recorded. *In vitro*, the role of sCD40L was studied on EC dysfunction and VSMC calcification after incubation with patients’ sera: cells engineered to knock down CD40 by siRNA were also used to confirm the role of CD40–CD40L pathway activation.

**Results:**

At study admission, the sCD40L median level of 8.4 ng/mL (interquartile range 2.9–12.7) showed the best statistical performance to identify MACE, which occurred in 51/201 (25.4%) patients. Indoxyl sulfate and p-cresyl sulfate directly correlated with sCD40L levels and induced its release by platelets. In comparison with PS, PMMA treatment significantly reduced sCD40L levels, in accordance with its enhanced mass removal by adsorption. *In vitro*, sera collected after PMMA treatment reduced EC dysfunction and VSMC osteoblastic differentiation through a mechanism involving the CD40–CD40L pathway.

**Conclusion:**

sCD40L is an independent predictor and mediator of MACE in chronic HD patients. PMMA membrane stably reduced sCD40L under the high-risk cut-off of 8.4 ng/mL. *In vitro* studies confirmed the role of PMMA in the reduction of EC dysfunction and VSMC calcification in association with sCD40L modulation.

## Graphical Abstract

**Graphical Abstract:**
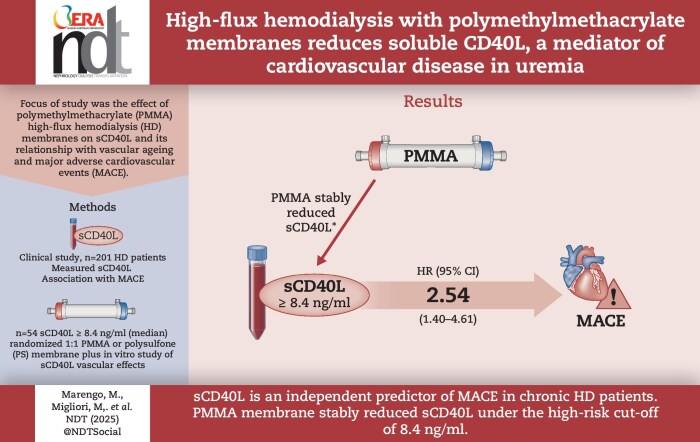

KEY LEARNING POINTS **What was known:**

- Major adverse cardiovascular events (MACE) are the main cause of mortality in chronic hemodialysis (HD) patients. Soluble CD40 ligand (sCD40L) interacts with the CD40 receptor located on vasculature, and the blockade of the CD40–CD40L pathway has a protective effect on endothelial cell (EC) and vascular smooth muscle cell (VSMC) dysfunction.
- The Japanese Registry showed a lower mortality when using polymethylmethacrylate (PMMA) membrane in high-flux HD.

**This study adds:**

- Median sCD40L (8.4 ng/mL) was higher than in previous studies and correlated with the levels of indoxyl sulfate/p-cresyl sulfate released by activated platelets.
- In comparison with polysulfone, PMMA membrane reduced sCD40L under the high-risk cut-off of 8.4 ng/mL through enhanced adsorption.
- PMMA inhibited EC and VSMC alterations induced by CD40–CD40L pathway activation.

**Potential impact:**

- The results of the present study suggest that sCD40L levels should be routinely measured in HD patients.
- The use of PMMA membrane should be considered in presence of sCD40L >8.4 ng/mL, in particular in patients not eligible for hemodiafiltration.
- Therapeutic interventions able to block CD40–CD40L pathway activation may limit MACE.

## INTRODUCTION

In 2021, the European Renal Association (ERA) Registry reported that the survival probability of incident hemodialysis (HD) patients 5 years after treatment start was 46.6%: mortality was 10–20 times higher than the age- and sex-matched general population, mainly due to the occurrence of major adverse cardiovascular events (MACE) [1, 2].

Inflammation and other non-traditional factors have a pivotal role in MACE development and in the accelerated aging processes of HD patients characterized by osteoporosis, sarcopenia and cognitive impairment [3–9].

The CD40–CD40 ligand (CD40L) costimulatory pathway plays a crucial role in the adaptive immune response. CD40, a 50-kDa membrane protein of the tumor necrosis factor family, is expressed by antigen presenting cells, whereas its agonist CD40L (CD154), a transmembrane 39-kDa protein, is highly expressed by activated T cells and platelets [10, 11]. Different vascular cells including endothelial cells (EC) and vascular smooth muscle cells (VSMC) express CD40 which is involved in the pathogenesis of atherosclerosis and calcifications. The soluble form of CD40L (sCD40L) is a 18-kDa functional trimer shed from activated cells that circulates in the bloodstream exerting biological effects on the vasculature: sCD40L is a negative prognostic factor among healthy individuals [12] and patients with acute coronary syndromes, and it is an independent predictor of re-stenosis after percutaneous transluminal angioplasty [13, 14].

The RISCAVID study previously demonstrated that serum sCD40L ≥7.6 ng/mL was associated with a significant increase of MACE in HD [15]: other studies with different follow-up periods showed similar results [16]. A higher expression of CD40 and CD40L was also observed in calcified plaques of HD patients [17]. Moreover, experimental data showed that the inhibition of the CD40–CD40L signaling through administration of an anti-CD40L monoclonal antibody protected from atherosclerotic lesions [18].

In 2017, the Japanese Dialysis Registry published that polymethylmethacrylate (PMMA) membrane reduced mortality after adjustments for dialysis-related variables and inflammation/nutrition parameters [19]. Due to its adsorptive properties [20], PMMA improves long-term clinical outcomes such as itching, carpal tunnel syndrome and joint pain [21, 22].

We herein designed a multicenter, observational and prospective study with the following primary endpoints: (i) to confirm the predictive role of sCD40L as a biomarker of MACE in HD; and (ii) to evaluate the role of PMMA in sCD40L modulation. As secondary endpoint, we studied *in vitro* PMMA-induced CD40–CD40L pathway modulation on EC dysfunction and VSMC calcification, key features of accelerated vascular aging of HD patients.

## MATERIALS AND METHODS

### Clinical studies

We enrolled 201 patients treated by high-flux bicarbonate HD in six Italian centers. Inclusion criteria were: age >18 and <75 years, HD from at least 6 months, vascular access with blood flow rate ≥250 mL/min. Exclusion criteria were: acute local or systemic infections, cancer, active autoimmune diseases and known allergy to polysulfone (PS) or polymethylmethacrylate (PMMA). The administration of nonsteroidal anti-inflammatory drugs and antibiotics was considered during the study period.

The local Ethical Committee of Azienda Sanitaria Locale (ASL) CN1 validated the protocol as principal investigator (ASL CN1/NEFRO 1-Ceva). All patients signed an informed consent and clinical studies were performed in accordance with the Declaration of Helsinki.

Samples were collected pre-dialysis in a fasting condition and kept frozen at –80°C until experimental use. Enzyme-linked immunosorbent assay (ELISA) for sCD40L serum levels (R&D Systems, Minneapolis, MN, USA) was performed in all patients and correlated with sex, age, dialysis vintage, causes of end-stage renal disease, and presence of cardiovascular risk factors including diabetes, hypertension, active smoking, alterations of lipid metabolism and previous MACE.

Serum levels of the protein-bound uremic toxins (PBUT) p-cresyl sulfate (pCS) and indoxyl sulfate (IS) were evaluated by liquid chromatography–mass spectrometry [23, 24] (see Supplementary Methods).

Considering the potential dropouts, 54/201 patients with sCD40L higher than the observed median level were randomized in two different groups and treated by high-flux HD using PS (Helixone^®^ series, Fresenius Medical Care, Bad Homburg, Germany) or PMMA membrane (BK-F series, Toray Industries Inc., Tokyo, Japan) with similar surfaces for nine consecutive months. The dialytic prescription (Qb, Qd, etc.) was not modified by switching from PS to PMMA and vice versa. Sample size was calculated based on the results of a preliminary study showing a reduction of 50% of sCD40L levels after 3 months of PMMA treatment [95% confidence interval (CI)].

Randomization was as follows: Group 1: PMMA (Months 0–3), PS (Months 3–6), PMMA (Months 6–9); Group 2: PS (Months 0–3), PMMA (Months 3–6), PS (Months 6–9).

At T0, T3, T6 and T9 (the number indicates months from start), blood specimens were obtained for central analysis and storage. The following variables were studied at all time points: Kt/V (Daugirdas formula), hemoglobin (Hb), hematocrit, C-reactive protein (CRP), transferrin saturation, ferritin, calcium, phosphate, parathyroid hormore (PTH), total proteins, β2-microglobulin, EPO Resistance Index [25]. Hepcidin serum levels were also evaluated by ELISA (DRG Instruments GmbH, Germany). All the variables were correlated with sCD40L levels at all time points.

Mass removal of sCD40L was also evaluated in two different HD sessions using PS or PMMA in 10 patients (see Supplementary Methods).

**Figure 1:**
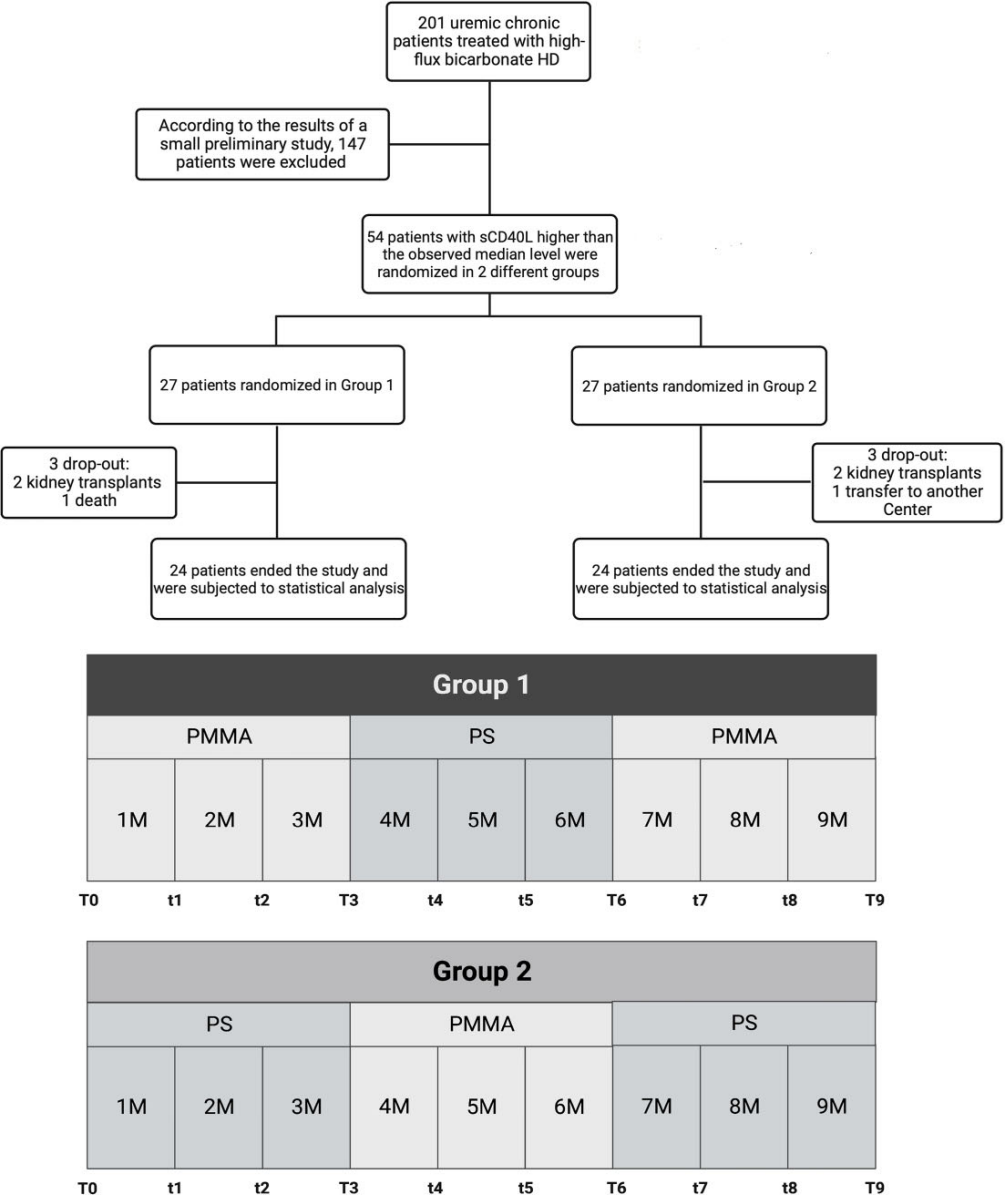
Clinical study design, flow-chart and randomization for different HD treatment. Upper panel: study design and flow-chart. Lower panel: randomization to Group 1 or Group 2 and HD treatment at different time points.

Clinical study design is described in Fig. 1.

### *In vitro* studies

#### Cell cultures

Platelets obtained from the blood bank were incubated with increasing doses of IS and pCS: supernatants were collected after 1 or 24 h and subjected to ELISA for sCD40L. Human umbilical vein-derived EC and VSMC were obtained by ATCC (Manassas, VA, USA) and cultured as previously described [5, 26]. Experiments on both cell types were performed using sera diluted 1:10 in culture medium [5, 6, 26]: in selected tests, EC and VSMC were engineered to knock-down CD40 by transfection with 80 pM of specific siRNA or an irrelevant control siRNA (Santa Cruz Biotechnology, Santa Cruz, CA, USA).

#### EC assays

The following tests were performed on EC incubated with patients’ sera: cell viability (XTT), fluorimetric evaluation of reactive oxygen species (ROS) production, flow cytometry analysis of adhesion molecules (E-selectin, ICAM-1) and of CD31, VE-cadherin, vimentin and type 1 collagen to detect endothelial-to-mesenchymal transition (EndMT), leukocyte adhesion, and quantitative reverse transcription polymerase chain reaction (qRT-PCR) for Nrf2 [27–29]. All the experimental procedures on EC are reported in Supplementary Methods.

#### VSMC assays

The following tests were performed on VSMC incubated with patients’ sera: cell calcification using red alizarin, qRT-PCR and fluorescence-activated cell sorting (FACS) analysis for RUNX2 expression [5, 26]. All the experimental procedures on VSMC are reported in Supplementary Methods.

### Statistical analyses

Statistical analyses were performed by SPSS statistical software v.17.0 (SPSS Inc., Chicago, IL, USA).

Differences between independent groups for continuous and categorical variables were estimated respectively by non-parametric Mann–Whitney U-test and Fisher's exact test (or Chi-square test).

Differences between time points were evaluated by non-parametric Friedman test and, if statistically significant (*P* < .05), differences between paired group (T0 vs T3, T3 vs T6, T6 vs T9) were evaluated by non-parametric Wilcoxon signed-rank test with Bonferroni's correction. Association between quantitative variables were evaluated by non-parametric Spearman test.

Differences in cardiovascular events between groups positive or negative for sCD40L in the follow-up period were evaluated by Kaplan–Meier analysis, Log-rank or Breslow test. Predictors for time to first cardiovascular event were evaluated by univariate and multivariate Cox regression analysis. Predictors for cardiovascular events within 2 years were also evaluated by logistic regression.

All data of *in vitro* experiments are expressed as average ± 1 SD. Statistical analysis was performed by analysis of variance and multiple comparison with ANOVA and Newmann–Keuls multicomparison test or Student's *t*-test where appropriated. *P*-values <.05 were considered as the threshold for statistical significance.

## RESULTS

### Clinical studies

**Figure 2:**
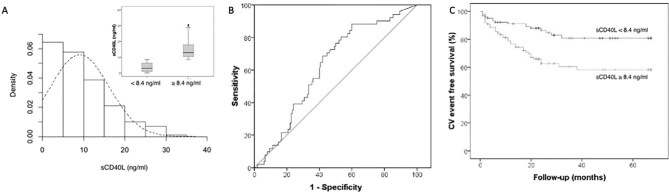
Distribution of sCD40L serum levels in enrolled patients. (**A**) Histograms showing sCD40L level distribution in all enrolled patients (*n* = 201). The inset depicts sCD40L levels in two subgroups divided according to the median level of 8.4 ng/mL. Boxes represent IQR with the median value shown as a horizontal bar within each box: bars outside each box show minimum and maximum values. (**B**) ROC curve for MACE observed during the follow-up according to sCD40L levels at study admission. (**C**) Kaplan–Meier curve showing cardiovascular event free survival in the two subgroups divided according to the median sCD40L level of 8.4 ng/mL.

We enrolled 201 HD patients using a PS membrane (Table 1): median sCD40L serum concentration at admission was 8.4 ng/mL [interquartile range (IQR) 2.9–12.7] (Fig. 2A). Using receiver operating characteristic (ROC) curves, we observed that the median level showed the best statistical performance to identify MACE in a follow-up period of 29 months (IQR 24–53 months) (Fig. 2B). MACE occurred in 51/201 (25.4%) patients; 33/201 (16.4%) died, 9/33 (27.2%) caused due to a proven cardiovascular event. We divided the cohort in two subgroups according to the sCD40L cut-off of 8.4 ng/mL, without finding any difference in clinical variables including MACE risk factors (Table 2). Through logistic regression analysis, we evaluated the correlation between sCD40L and MACE: multivariate Cox regression analysis confirmed that age and sCD40L were independent risk factors for MACE (Table 3). Kaplan–Meier curve showing the significant difference of MACE-free survival between the two groups is reported in Fig. 2C.

**Table 1:** Baseline demographic and clinical features of the 201 enrolled patients.

	Median (IQR) or number (percentage)
Age at study enrollment (years)	68 (57–74)
Age at the initiation of dialysis treatment (years)	64 (53–71)
Dialysis vintage (months)	28 (14–56.5)
Male	111 (55)
Diabetes	84 (42)
Hypertension	167 (83)
Dyslipidemia	78 (39)
Smoking	74 (37)
Previous MACE	80 (40)

Continuous data are presented as median (IQR), while categorical data as absolute number (percentage).

**Table 2:** Comparison of the main traditional cardiovascular risk factors in patients with sCD40L levels <8.4 or ≥8.4 ng/mL.

	sCD40L <8.4 ng/mL (*n* = 100)	sCD40L ≥8.4 ng/mL (*n* = 101)	*P*-value
Age (years)	68 (56–75)	68 (60.75–74)	.822
Dialysis vintage (months)	30 (14–60)	26 (14.75–50)	.577
Male	56 (56)	54 (53.1)	.644
Diabetes	38 (38)	47 (46.9)	.193
Hypertension	81 (81)	88 (87.6)	.174
Dyslipidemia	38 (38)	41 (40.8)	.668
Smoking	34 (34)	40 (39.8)	.393
Previous MACE	36 (36)	44 (43.9)	.249

Continuous data are presented as median (IQR), while categorical data as absolute number (percentage).

**Table 3:** Univariate and multivariate Cox proportional hazard regression analyses of risk factors for MACE development.

	HR (95% CI)	*P*-value
Univariate Cox proportional hazard regression analysis		
sCD40L ≥8.4 ng/mL	2.677 (1.480–4.841)	.001*
Age	1.030 (1.005–1.055)	.017*
Dialysis vintage	0.999 (0.993–1.004)	.672
Male	1.416 (0.806–2.488)	.227
Diabetes	1.811 (1.04–3.153)	.036*
Hypertension	2.384 (0.858–6.625)	.096
Dyslipidemia	1.123 (0.253–1.962)	.683
Smoking	1.036 (0.587–1.828)	.902
Previous MACE	1.650 (0.952–2.859)	.074
IS	0.992 (0.982–1.002)	.101
pCS	0.992 (0.984–1.001)	.092
Multivariate Cox proportional hazard regression analysis		
sCD40L ≥8.4 ng/mL	2.542 (1.403–4.606)	.002*
Diabetes	1.567 (0.899–2.733)	.113
Age	1.031 (1.004–1.058)	.010*

HR, hazard ratio.

**Figure 3:**
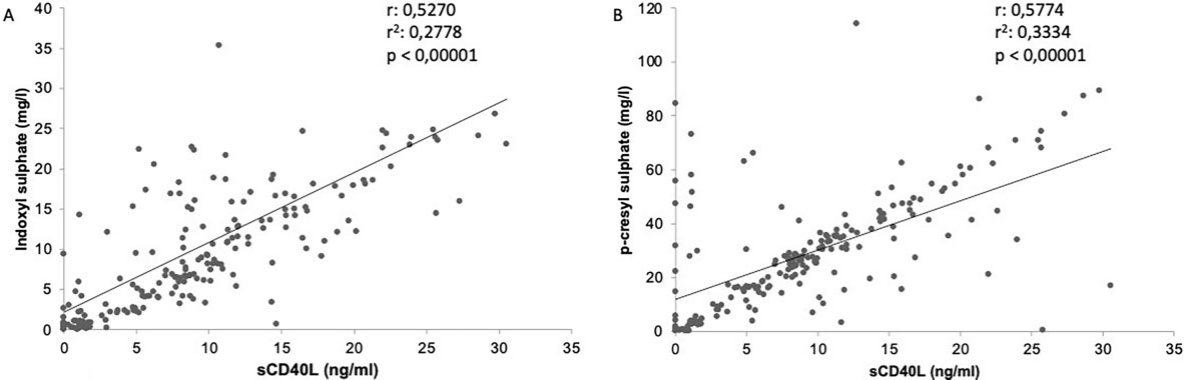
Correlation between sCD40L serum levels and PBUT. (**A**) Linear correlation between sCD40L (ng/mL) and IS (expressed in mg/L): r = 0.5270, r^2^ = 0.2778, *P* < .00001. (**B**) Linear correlation between sCD40L (ng/mL) and pCS (expressed in mg/L): r = 0.5774, r^2^ = 0.3334, *P* < .00001.

**Figure 4:**
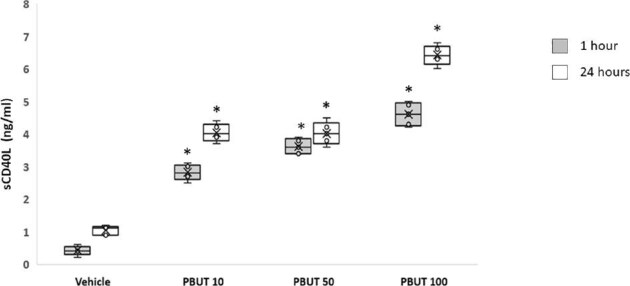
*In vitro* evaluation of sCD40L release from human platelets incubated with PBUT. Increasing doses (PBUT 10, 50 or 100 µg/mL) of IS and pCS induced a significant release of sCD40L assessed by ELISA after 1 h (gray plots) or 24 h (white plots); (*P < .05 PBUT 10, 50 or 100 µg/mL vs vehicle at both h and 24 h). Data are expressed as average ± 1 SD of five different experiments.

We also found a linear correlation between sCD40L and IS or pCS (Fig. 3A and B, respectively). Therefore, we investigated *in vitro* the biological effects of increasing doses (10, 50 or 100 μg/mL) of IS and pCS on platelets: PBUT induced an enhanced release of sCD40L from platelets 1 h and 24 h after incubation (Fig. 4).

**Figure 5:**
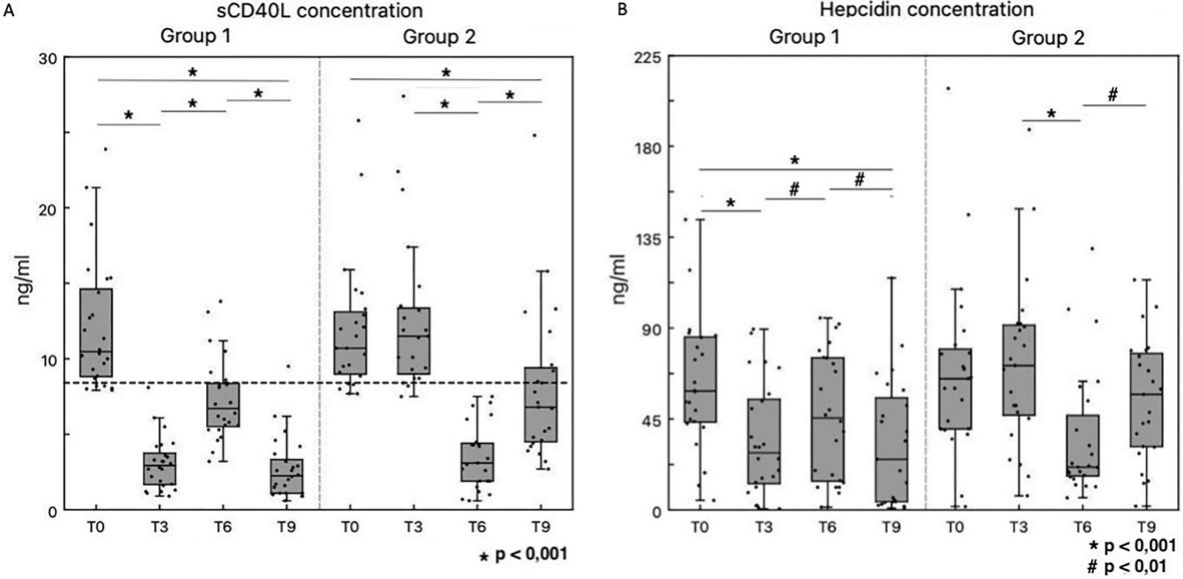
Modulation of sCD40L and Hepcidin serum levels. (**A**) ELISA showing sCD40L serum levels at different time points (T0, T3, T6, T9) in Group 1 (left panel) and Group 2 (right panel). Each 3-month treatment with PMMA induced a significant decrease of sCD40L (Group 1: *P < .001 T3 vs T0 and T9 vs T6; Group 2: *P < .001 T6 vs T3). By contrast, each 3-month treatment with PS after PMMA was associated with a significant increase of sCD40L (Group 1: *P < .001 T6 vs T3; Group 2: *P < .001 T9 vs T6). No significant difference was observed in Group 2 between T0 and T3 during PS treatment. In both groups a significant difference of sCD40L between T0 and T9 was observed (Group 1 and 2: *P < .001 T9 vs T0): in addition, in both groups, sCD40L at T9 was lower than the cut-off risk of 8.4 ng/mL (bold dotted line). (**B**) ELISA showing Hepcidin serum levels at different time points (T0, T3, T6, T9) in Group 1 (left panel) and Group 2 (right panel). Each 3-month treatment with PMMA induced a significant decrease of Hepcidin (Group 1: *P < .001 T3 vs T0, ^#^*P* < .01 T9 vs T6; Group 2: *P < .001 T6 vs T3). By contrast, each 3-month treatment with PS after PMMA was associated with a significant increase of Hepcidin (Group 1: ^#^*P* < .01 T6 vs T3; Group 2: ^#^*P* < .01 T9 vs T6). No significant difference was observed in Group 2 between T0 and T3 during PS treatment. In Group 1 but not in Group 2, a significant difference of Hepcidin levels between T0 and T9 was observed (*P < .001 T9 vs T0). Wilcoxon signed ranks test was used for statistical analysis.

Our main working hypothesis was that PMMA membrane may modulate sCD40L serum levels: for this purpose, 54/201 patients with sCD40L ≥8.4 ng/mL were enrolled in a crossover study and randomized to Group 1 (*n* = 27) or Group 2 (*n* = 27) (Fig. 1). We recorded six drop-outs (four transplants, one death, one transfer) and 48 patients reached the end of the study after 9 months (*n* = 24 in each group). At T0, patients showed the following parameters (average ± 1 SD): CRP 4.90 ± 6.28 mg/L, fibrinogen 350.02 ± 109 mg/L, β2-microglobulin 16.87 ± 8.32 mg/L, albumin 3.50 ± 0.05 g/dl, Hb 10.5 ± 1.2 g/dl, PTH 378.3 ± 300 pg/mL and Kt/V 1.48 ± 0.22. The average serum levels of sCD40L was 12.11 ± 3.32 ng/mL. We did not observe any significant difference of these variables at all time-points in either group, except for sCD40L and Hepcidin. Wilcoxon signed ranks test showed that each 3-month treatment with PMMA significantly reduced sCD40L levels: the difference of sCD40L between T0 and T9 was statistically significant in both groups, always under the cut-off risk of 8.4 ng/mL (Fig. 5A). Similar results were observed for Hepcidin: however, a significant difference between T0 and T9 was found only in Group 1 (Fig. 5B). A trend of CRP decrease without reaching statistical significance was observed in particular in Group 1. Since albumin variation may influence PBUT levels, we also evaluated albumin at all time points without finding any significant difference.

**Figure 6:**
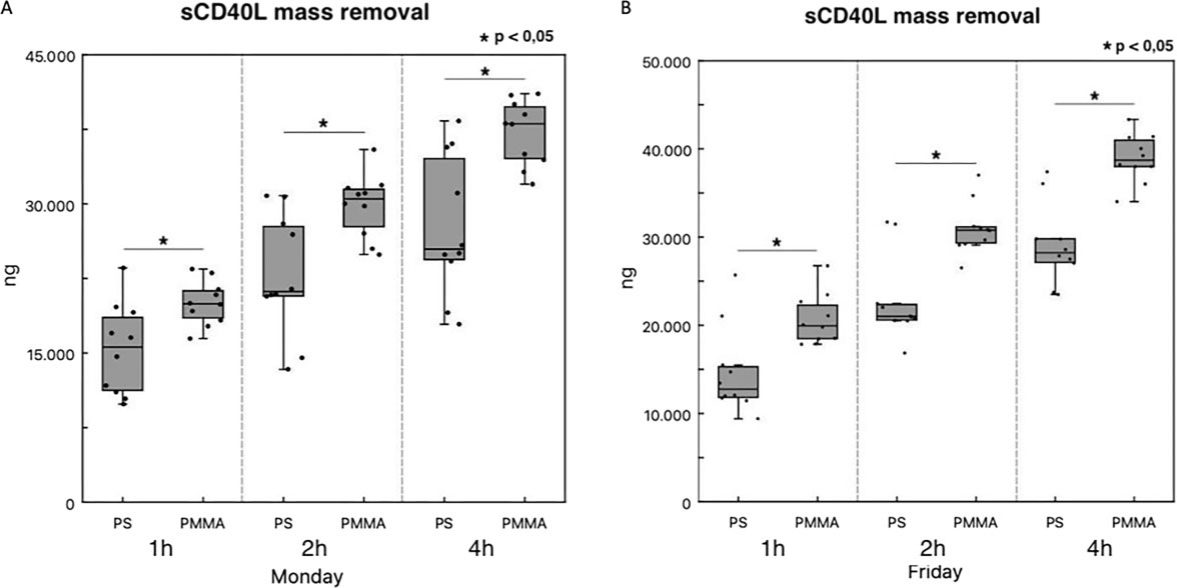
Evaluation of sCD40L mass removal with different membranes. ELISA showing sCD40L serum levels in 10 patients enrolled in Group 2. Data are expressed as average ± 1 SD in two different dialysis sessions of the same week (**A**, Monday; **B**, Friday). In comparison with PS, PMMA was associated with a significant higher sCD40L mass removal at all time points considered in both HD sessions (*P < .05 PMMA vs PS at 1 h, 2 h and 4 h).

In 10 patients of Group 2, we performed the analysis of sCD40L mass removal in two different HD sessions, comparing PS and PMMA in two consecutive weeks. We observed a significant higher sCD40L mass removal by using PMMA at all time points (Fig. 6A and B).

### *In vitro* studies

All *in vitro* studies on EC and VSMC were performed using sera collected at different time points from Group 1 (*n* = 24).

#### EC assays

**Figure 7:**
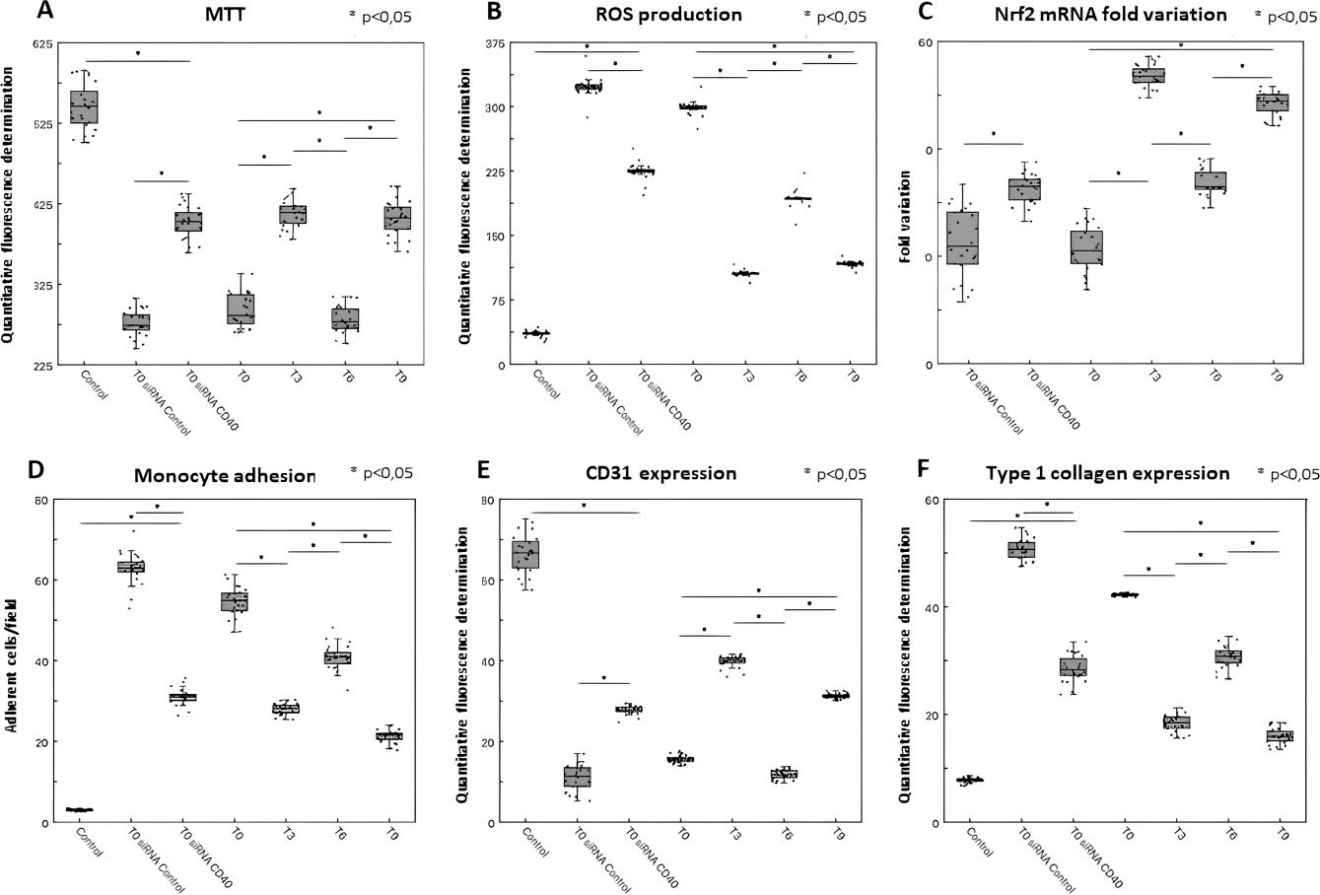
*In vitro* assessment of EC viability, ROS production, *Nrf2* mRNA expression, monocyte adhesion and EndMT. All *in vitro* studies on EC were performed using sera collected at different time points from Group 1 (*n* = 24). (**A**) Wild-type, siRNA control and siRNA CD40 EC viability evaluated by MTT assay after 96 h incubation with sera collected at different time points (results are expressed as quantitative fluorescence determination in *y*-axis). PMMA treatment significantly reduced uremic sera-induced EC cytotoxicity (*P < .05 T3 vs T0 and T9 vs T6). PS treatment after PMMA induced a significant decrease of EC viability (*P < .05 T6 vs T3). A significant increase of EC viability was also observed between T0 and T9 (*P < .05 T9 vs T0). A significant cell viability augmentation was observed in siRNA CD40 EC but not in siRNA control EC challenged with T0 sera (*P < .05 siRNA CD40 EC vs siRNA control). Despite the decrease of cytotoxicity observed by down-regulating the CD40 receptor, siRNA CD40 EC viability was significantly lower than that observed with control (*P < .05 siRNA CD40 EC vs control). (**B**) ROS production by wild-type, siRNA control and siRNA CD40 EC incubated with sera collected at different time points (results are expressed as quantitative fluorescence determination in *y*-axis). PMMA treatment significantly reduced ROS production (*P < .05 T3 vs T0 and T9 vs T6). PS treatment after PMMA induced a significant increase of ROS production (*P < .05 T6 vs T3). A significant decrease of ROS production was also observed between T0 and T9 (*P < .05 T9 vs T0). By contrast, a significant ROS reduction was observed in siRNA CD40 EC but not in siRNA control EC challenged with T0 sera (*P < .05 siRNA CD40 EC vs siRNA control). However, ROS generation by siRNA CD40 EC was significantly higher than that observed with control (*P < .05 siRNA CD40 EC vs control). (**C**) *Nrf2* mRNA expression in wild-type, siRNA control and siRNA CD40 EC incubated with sera collected at different time points (results are expressed as fold variation with respect to control). PMMA treatment significantly enhanced Nrf2 expression (*P < .05 T3 vs T0 and T9 vs T6). PS treatment after PMMA induced a significant reduction of Nrf2 expression (*P < .05 T6 vs T3). A significant increase of Nrf2 expression was also observed between T0 and T9 (*P < .05 T9 vs T0). A significant *Nrf2* mRNA augmentation was also detected in siRNA CD40 EC vs siRNA control EC (*P < .05 siRNA CD40 EC vs siRNA control EC). (**D**) Monocyte adhesion to wild-type, siRNA control and siRNA CD40 EC after incubation with sera collected at different time points (results are expressed as number of adherent cells/microscopic field). PMMA treatment significantly reduced monocyte adhesion (*P < .05 T3 vs T0 and T9 vs T6). PS treatment after PMMA significantly increased monocyte adhesion (*P < .05 T6 vs T3). A significant reduction of monocyte adhesion was also observed between T0 and T9 (*P < .05 T9 vs T0). A significant decrease of monocyte adhesion was found in siRNA CD40 EC but not in siRNA control EC challenged with T0 sera (*P < .05 siRNA CD40 EC vs siRNA control). Despite the decrease of inflammatory cell binding observed by down-regulating the CD40 receptor, monocyte adhesion to siRNA CD40 EC was significantly higher than that observed with control (*P < .05 siRNA CD40 EC vs control). (E, F) EndMT characterization by FACS analysis for the endothelial antigen CD31 (**E**) and for the fibroblast molecule type 1 collagen (**F**) in wild-type, siRNA control and siRNA CD40 EC incubated with sera collected at different time points (results are expressed as quantitative fluorescence determination in *y*-axis). PMMA treatment significantly reduced EndMT [*P < .05 T3 vs T0 and T9 vs T6 for CD31 increase in (E) and for type 1 collagen decrease in (F)]. PS treatment after PMMA significantly increased EndMT [*P < .05 T6 vs T3 for CD31 decrease in (E) and for type 1 collagen increase in (F)]. A significant decrease of EndMT was also observed between T0 and T9 [*P < .05 T9 vs T0 for CD31 increase in (E) and for type 1 collagen decrease in (F)]. A significant EndMT reduction was also observed in siRNA CD40 EC but not in siRNA control EC challenged with T0 sera [*P < .05 siRNA CD40 EC vs siRNA control for CD31 increase in (E) and for type 1 collagen decrease in (F)]. EndMT in siRNA CD40 EC was significantly higher than that observed with control [*P < .05 siRNA CD40 EC vs control for CD31 decrease in (E) and for type 1 collagen increase in (F)].

A dose-dependent experiment was performed to identify the optimal percentage of sera able to induce 50% reduction of EC survival: 10% sera induced this effect (not shown) and we then adopted this dose for all assays. In comparison with control, EC viability was decreased in the presence of T0 sera. By contrast, when compared with T0, incubation with T3 sera resulted in a significant increase of cell viability. EC injury newly increased after stimulation with T6 sera and then decreased again with T9 sera (Fig. 7A). The role of the CD40–CD40L pathway on EC cytotoxicity was studied using cells engineered to knock-down CD40 by specific siRNA: T0 sera-induced cytotoxicity was significantly decreased in siRNA CD40 EC in comparison with siRNA control or wild-type EC (Fig. 7A). Similar results were obtained for ROS production (Fig. 7B). This protective effect on endothelial dysfunction was confirmed by qRT-PCR, showing an increased expression of mRNA coding for the antioxidant molecule Nrf2 in wild-type EC treated with sera collected after PMMA treatment or in siRNA CD40 EC challenged with T0 sera (Fig. 7C). Moreover, incubation with sera collected after PMMA treatment induced a significant reduction of monocyte adhesion (Fig. 7D) with a concomitant down-regulation of adhesion molecule expression on EC (E-selectin, ICAM-1) (not shown). A similar decrease of monocyte adhesion was observed in siRNA CD40 EC but not in control siRNA EC incubated with T0 sera (Fig. 7D). Furthermore, EndMT, defined as the loss of endothelial phenotype (expression of CD31 in Fig. 7E) with transformation into fibroblast (expression of type 1 collagen in Fig. 7F), was reduced incubating EC with sera collected after PMMA treatment, suggesting a limitation of uremic sera-induced cellular senescence. A significant reduction of EndMT was also detected in siRNA CD40 EC but not in siRNA control EC (Fig. 7E and F).

#### VSMC assays

**Figure 8:**
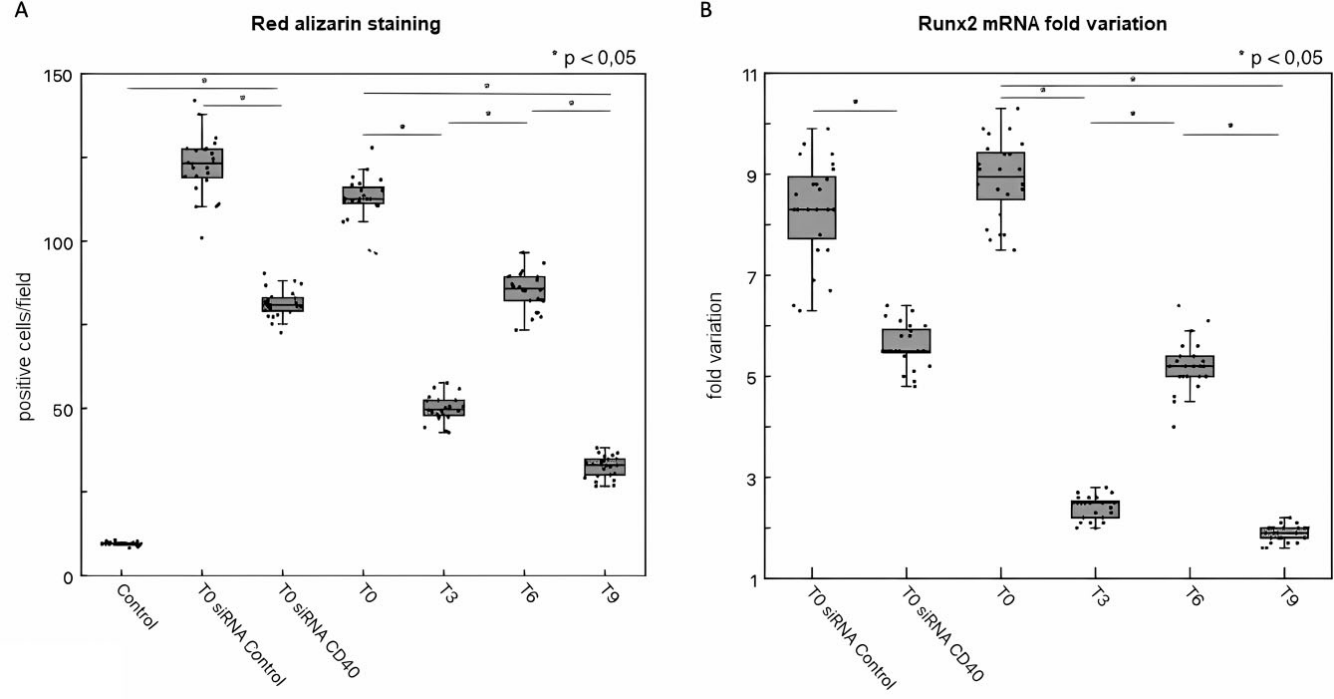
Evaluation of *in vitro* calcification and *Runx2* mRNA expression of VSMC. All *in vitro* studies on VSMC were performed using sera collected at different time points from Group 1 (*n* = 24). (**A**) Red alizarin staining of wild-type, siRNA control and siRNA CD40 VSMC incubated with sera collected at different time points (results are expressed as number of positive cells/microscopic field). PMMA treatment significantly reduced VSMC calcification (*P < .05 T3 vs T0 and T9 vs T6). PS treatment after PMMA induced a significant increase of red alizarin staining (*P < .05 T6 vs T3). A significant decrease of VSMC calcification was also observed between T0 and T9 (*P < .05 T9 vs T0). By contrast, a significant red alizarin staining reduction was observed in siRNA CD40 VSMC but not in siRNA control VSMC challenged with T0 sera (*P < .05 siRNA CD40 vs siRNA control). Red alizarin staining of siRNA CD40 VSMC was significantly higher than that observed with control (*P < .05 siRNA CD40 VSMC vs control). (**B**) Runx2 expression in wild-type, siRNA control and siRNA CD40 VSMC incubated with sera collected at different time points (results are expressed as fold variation with respect to control). PMMA treatment significantly reduced Runx2 expression (*P < .05 T3 vs T0 and T9 vs T6). PS treatment after PMMA induced a significant increase of Runx2 expression (*P < .05 T6 vs T3). A significant decrease of Runx2 expression was also observed between T0 and T9 (*P < .05 T9 vs T0). A significant *Runx2* mRNA reduction was detected in siRNA CD40 VSMC vs siRNA control VSMC incubated with T0 sera (*P < .05 siRNA CD40 vs siRNA control).

In comparison with control, incubation of VSMC with T0 sera enhanced their calcification processes as assessed by red alizarin staining (Fig. 8A). A significant reduction of calcium deposition incubating VSMC with sera drawn after PMMA, but not PS, treatment was observed. Compared with siRNA control VSMC, red alizarin staining was significantly lower in siRNA CD40 VSMC (Fig. 8A). Similar results were observed for *Runx2* mRNA expression (Fig. 8B), suggesting that sCD40L removal by PMMA may limit their osteoblastic differentiation.

## DISCUSSION

In this study, we confirmed that the soluble form of the costimulatory molecule CD40 ligand (sCD40L) is an independent predictor of MACE in HD patients. We found that sCD40L ≥8.4 ng/mL identifies HD patients at high risk for MACE. In a second part of the clinical study, we observed the potential protective role of PMMA on MACE in association with the decrease of sCD40L due to its enhanced intradialytic removal. *In vitro* studies on EC and VSMC confirmed that PMMA may limit the activation of the CD40–CD40L pathway involved in endothelial dysfunction and vascular calcification. Last, we identified a direct correlation between sCD40L and IS/pCS serum levels and *in vitro* studies showed that these PBUT enhanced sCD40L release from activated platelets.

Inflammation and oxidative stress are non-traditional causes of premature vascular aging and MACE in HD patients. In this setting, the activation of the CD40–CD40L pathway may play a crucial role in the inflammatory response and in the triggering of vascular damage, since both CD40 and CD40L are highly expressed in calcified and non-calcified vascular lesions [17]. In the RISchio CArdiovascolare nei pazienti afferenti all’ Area Vasta In Dialisi (RISCAVID) study, a value of sCD40L ≥7.6 ng/mL was identified as the cut-off for a higher risk for MACE. We herein found that sCD40L ≥8.4 ng/mL was an independent predictor of MACE during a follow-up period of 29 months. In comparison with RISCAVID, we observed a lower dialysis vintage and a higher incidence of diabetes and hypertension, reflecting the phenotypic changes occurred in the HD population over the last 15 years. Despite a similar percentage of previous MACE and age/sex distribution, with respect to RISCAVID, we found a higher prevalence of diabetes (42% vs 19%) and hypertension (83% vs 62%), and a lower dialysis duration (28 vs 46 months). Furthermore, 28% of RISCAVID patients were treated by hemodiafiltration; conversely, all patients in the present study were treated by high-flux HD. The multivariate COX regression analysis confirmed that median age and sCD40L were independent risk factors for MACE.

Several studies demonstrated that PBUT are barely removed by standard HD techniques [30] and are mediators of EC [31, 32] and VSMC damage [33]. We studied the relationship between IS/pCS and sCD40L, finding a direct correlation of both PBUT with sCD40L and *in vitro* experiments confirmed that IS and pCS can directly induce sCD40L release from platelets. PMMA has been associated with better survival rates [19, 34, 35] due to an enhanced adsorption of middle and high molecular weight molecules [35, 36].

Our main working hypothesis was that PMMA could modulate sCD40L levels. For this purpose, 54 patients with sCD40L ≥8.4 ng/mL were randomized to two groups alternatively treated by PMMA or PS. Serum levels of sCD40L were significantly reduced under the high-risk cut-off of 8.4 ng/mL each time that PMMA was used for at least 3 months. Moreover, we observed a significant decrease of Hepcidin only in Group 1 at the end of the observational period, suggesting that at least 6 months of PMMA treatment was necessary to obtain this result. A non-significant reduction of CRP was also observed in both groups. We could speculate that these results might indicate a potential modulatory effect of PMMA on inflammatory parameters correlated with MACE and anemia. However, further studies enrolling a larger number of patients and using PMMA for a longer period are needed to confirm this hypothesis.

The beneficial effects of PMMA on sCD40L levels could be ascribed to its intradialytic removal. In comparison with PS, we observed a significant higher sCD40L mass removal with PMMA. Convective driven strategies such as hemodiafiltration can remove sCD40L: however, a limited percentage of patients can reach the recommended high convective volume due to an inadequate blood flow rate. In this clinical setting, adsorptive membranes such as PMMA used in high-flux HD modality could improve sCD40L removal. Finally, we performed *in vitro* experiments aimed to evaluate PMMA-induced modulation of the CD40–CD40L pathway on EC and VSMC. The inflammatory microenvironment created by the uremic milieu leads to an increased expression of CD40 with different functional consequences correlated with early vascular aging [37]. CD40 activation on EC leads to an increased expression of adhesion molecules (ICAM-1, VCAM-1, E-selectin) and to the production of inflammatory cytokines that sustain senescence-associated secretory phenotype [38]. In VSMC, the activation of the CD40–CD40L pathway induces an increased expression of metalloproteases (MMPs), stromelysin (MMP-11) and inflammatory cytokines [39]. Taken together, these results suggested that CD40 signaling is crucial for atherosclerotic plaque instability, EC dysfunction/senescence and VSMC osteoblastic differentiation. Previous experimental data showed that the administration of a blocking monoclonal antibody directed to CD40 reduces atherosclerotic lesions [39]. We herein performed experiments using sera from patients treated by PMMA or PS on wild-type EC and VSMC or on the same cells engineered to knock-down CD40 by siRNA. PMMA-treated sera improved EC viability and reduced adhesion of inflammatory cells: this protective effect may be ascribed to a decreased intracellular production of ROS and to the inhibition of senescence processes such as EndMT and the preserved expression of Nrf2. Recent studies demonstrated that EndMT is one of the main drivers of atherosclerosis progression [40]. In addition, intracellular levels of Nrf2, a master regulator of the antioxidant response, are decreased in HD patients: current findings suggest that Nrf2 agonists may represent a novel target for the protection against vascular senescence and calcifications [41–44]. We herein demonstrated that after PMMA treatment, HD sera reduced their ability to trigger EndMT and to reduce Nrf2 levels in EC. These biological effects seem to be at least in part due to a reduced activation of the CD40–CD40L pathway, since the incubation of siRNA CD40 EC with sera obtained at T0 reduced cell dysfunction. Similar results were observed in VSMC: PMMA-treated sera reduced calcium deposition detected by red alizarin staining and mRNA coding for Runx2, a key factor for VSMC osteoblastic differentiation, with a mechanism dependent on CD40–CD40L activation.

We acknowledge that our study presents some weaknesses: the clinical study enrolled a small number of patients and the treatment with PMMA was limited to 6 months in Group 1 and 3 months in Group 2. However, the 54 enrolled patients represented a significant sample of the whole population regarding age, sex, dialysis vintage and risk factors for MACE such as diabetes, hypertension, dyslipidemia and smoking. Several other comorbidities (heart failure, obesity, chronic obstructive pulmonary disease, etc.) that may influence sCD40L levels have not been evaluated in this study. Based on sCD40L molecular weight, we hypothesized that its removal by PMMA during high-flux HD could be mainly ascribed to adsorption; however, these results should be confirmed by sCD40L measurement in the dialysate that was not performed. Moreover, the modulation of inflammatory parameters may be ascribed not only to uremic toxins adsorption, but also to the higher content of polyvinylpyrrolidone and bisphenol A in PS compared with PMMA membrane [45, 46]. This study also presents some strengths: we expanded the results of previous clinical trials showing that sCD40L is an independent biomarker of MACE. For the first time, we demonstrated that PMMA can reduce sCD40L levels, as well as other inflammatory biomarkers such as Hepcidin. In addition, *in vitro* studies confirmed the prominent role of the CD40–CD40L pathway in EC dysfunction and VSMC calcification: PMMA removal of sCD40L may limit these detrimental effects responsible for an earlier vascular aging.

In conclusion, PMMA membrane reduces serum levels of sCD40L, an independent predictor and mediator of MACE in chronic HD patients. Further studies performed in a larger cohort of patients and aimed to evaluate long-term clinical effects of PMMA are needed to confirm our results that may partially explain the encouraging data on survival reported in dialysis registries.

## Supplementary Material

gfaf101_Supplemental_File

## Data Availability

Clinical data are available in the informatics databases of the Dialysis Centers involved in the study. Laboratory data are retained at the Department of Translational Medicine (DIMET) of the University of Piemonte Orientale (UPO)-UPO Aging Registry.
