# Supplementary material for: High-flux hemodialysis with polymethylmethacrylate membranes reduces soluble CD40L, a mediator of cardiovascular disease in uremia
Source: Nephrol Dial Transplant. 2025 Jun 6;40(11):2131–42. doi: 10.1093/ndt/gfaf101 (PMC12559792; doi:10.1093/ndt/gfaf101)
Supplement: gfaf101_Supplemental_File [file gfaf101_supplemental_file.docx]

**Polymethylmethacrylate membrane reduces soluble CD40-Ligand, an independent predictor and mediator of cardiovascular events in chronic hemodialysis patients.**

**Supplementary Methods**

**Determination of p-cresyl sulfate (pCS) and indoxyl sulfate (IS) serum levels by Liquid Chromatography-Mass Spectrometry (LCMS)**

Serum levels of the protein-bound uremic toxins (PBUT) p-cresyl sulfate and indoxyl sulfate were evaluated by Liquid Chromatography-Mass Spectrometry (LCMS). Briefly, aliquots of 100 μL of plasma were thawed and spiked with D7 p-cresyl sulfate potassium salt and 13C6 indoxyl sulfate potassium salt as internal standard (IS) compounds, adding 300 μL of cold methanol. The samples were centrifuged 8000 g for 10 minutes at 4°C and the supernatants were diluted using HPLC mobile phase. The chromatographic separations were run on a Phenomenex Gemini C18 100 Å column, 150 × 2.0 mm, 1.7 μm particle size (Phenomenex, Bologna, Italy), thermostated at 40°C. Injection volume was 3 μL and flow rate 300 μL/min. Gradient mobile phase composition was adopted: 5:95 to 95:5 methanol/aqueous 5 mM ammonium acetate in 15 min. A Shimadzu Nexera (Shimadzu, Kyoto, Japan) X2 UPLC coupled with a Sciex 5500 Q-trap mass spectrometer (Sciex, Framingham, MA, USA) equipped with a Turbo Ion Spray atmospheric pressure interface (ESI ion source) was used. The LC column effluent was delivered into the ion source using nitrogen as sheath and auxiliary gas. The ion source temperature was set at 550°C and the needle voltage at the -4.5 kV value. The acquisition method used was previously optimized in the tuning sections for the analyte ion (capillary, magnetic lenses and collimating multipoles voltages) in order to achieve the maximal sensitivity. Spectra were acquired in the negative ion mode. The detector was used in MRM mode and the transitions for uremic toxins and IS detection were: 187 to 107, 80 *m/z*, collision energy (CE) -30, and -32 V respectively; 194 to 114, 80 *m/z* (CE -26 and - 21 V); 212 to 132, 80 *m/z* (CE -24 V); 218 to 138, 80 *m/z* (CE -25 and - 24 V). A calibration curve was created using standard solutions of the analytes, finding lower limits of quantification (LLOQ) of 20 μg/Kg for indoxyl sulfate and 0.5 μg/Kg for p-cresyl sulfate. The method was validated in order to check selectivity, linearity, accuracy and precision following the FDA guidelines (references 23, 24 main text).

**sCD40L Mass Removal evaluation**

Mass Removal (MR) of sCD40L was also evaluated in 2 different HD sessions using PS or PMMA in 10 patients with the following formula:

*sCD40L MR= [Body Volume before HD (ml) x sCD40L before HD (ng/ml)] - [Body Volume after 1-2 or 4 hrs HD (ml) x sCD40L after 1-2 or 4 hrs HD (ng/ml)]*

where:

- Body Volume before HD (ml) = 1000 x Body Weight before HD (Kg) x (1/13)

- Body Volume after 1-2 or 4 hrs HD (ml) = 1000 x Body Weight before HD (Kg) x (1/13) x [Hct before HD (%)/Hct after 1-2 or 4 hr HD (%)]

***In vitro* studies on Endothelial Cells (EC)**

*Cell viability*: EC were cultured on 24-well plates (Falcon Labware, Oxnard, CA) at a concentration of 5 × 10^4^ cells/well and incubated with sera drawn at different time points in presence of 250 μg/ml XTT (Sigma, St. Louis, MO) in a medium lacking phenol red. The absorption values at 450 nm were measured in an automated spectrophotometer at different time points.

*Reactive Oxygen Species production*: Reactive Oxygen Species (ROS) production was assessed by Image–iT® Detection Kit (Life Technologies, Carlsbad, CA). The assay is based on 5-(and-6)-carboxy2′,7′-dichlorodihydrofluorescein diacetate (carboxyH2 DCFDA), a compound that releases fluorescence after binding ROS. Experiments were conducted according to the manufacturer’s instructions: after incubation with selected stimuli, cells were analyzed under a fluorescence microscope or re-suspended with EDTA and analyzed by FACS.

*Endothelial-to-mesenchymal transition (EndMT):* EC phenotype was analyzed by surface staining with Allophycocyanin (*APC*)-conjugated anti-CD31 (Miltenyi Biotec B.V. & Co. KG, Germany) Phycoerythrin (*PE*)-conjugated anti-VE-cadherin (Miltenyi Biotec), and intracellular staining with *PE*-conjugated anti-Vimentin antibody (Miltenyi Biotec) and Fluorescein isothiocyanate (*FITC*)-conjugated anti-Collagen I antibody (Merck Millipore, Burlington, MA). Briefly, after incubation, EC were washed and detached by ice cold 1x PBS EDTA. For surface staining, EC were re-suspended in PBS pH 7.2, 0.2% bovine serum albumin, and 0.02% sodium azide and then incubated with FCR blocking reagent (Miltenyi Biotec) for 10 min at room temperature. After blocking incubation, surface markers were added for 15 min at 4°C. EC were then washed, re-suspended in FACS buffer and analyzed. For intracellular staining, cells were permeabilized by Intraprep reagents (Instrumentation Laboratory S.p.A. - Werfen, Milan, Italy), then incubated with FCR blocking reagent (Miltenyi Biotec) for 10 min and with intracellular antibodies for 20 minutes at 4°C. Finally, EC were washed and re-suspended in FACS buffer for acquisition. Data were obtained using a FC500 (Beckmann Coulter, Brea, CA) flow cytometer and analyzed: the area of positivity was determined using an isotype-matched mAb (reference 27 main text).

*Leukocyte adhesion:* Peripheral Blood Mononuclear Cells (PBMC) were obtained from healthy volunteers after isolation with Ficoll Hypaque (GE Healthcare, Chicago, IL) density gradient and added to EC monolayers in presence of different stimuli as previously described (references 28, 29 main text). PBMC were then labeled for 15 minutes with 10 µM of fluorescent Vybrant Cell Tracer kit (Life Technologies, Carlsbad, CA) in RPMI. Fluorescent cells were resuspended in EBM without FCS (50 × 10^6^ /ml) and added to confluent monolayer of EC on six-well plates. After 1hr at 37°C of slight agitation, samples were washed, fixed with 4% paraformaldehyde and observed under a UV light microscope. Fluorescent cells were quantified in 10 different fields at x200 magnification. The expression of the adhesion molecules E-selectin and ICAM-1 was evaluated by FACS using appropriate antibodies (Santa Cruz Biotechnology).

*Quantitative RT-PCR for Nrf2 expression*: total RNA was extracted by EC after appropriate stimulation using mirVana RNA isolation kit (Life Technologies). RNA concentration and purity were detected by the NanoDrop1000 spectrophotometer. We evaluated Nrf2 mRNA expression by using High cDNA Reverse Transcription Kit (Applied Biosystems, Foster City, CA) and the Power SYBR Green PCR Master Mix on a 96-well StepOnePlus Real Time System (Applied Biosystems, Waltham, MA). Actin-β was used as housekeeping gene. Fold change in RNA expression was calculated as 2-ΔΔCt using the geometric mean in Ct values as normalizer. The following primers were used: • Actin-β: forward, 5’-GAG TCC GGC CCC TCC AT-3’; reverse; 5’-GCA ACT AAG TCA TAG TCC GCC TAG A-3’ • Nrf2: forward 5'-TCT GAC TCC GGC ATT TCA CT -3'; reverse 5'-GGC ACT GTC TAG CTC TTC CA -3'.

*RNA interference for CD40:* in selected experiments, EC were seeded on 24-well plates and engineered to knock-down CD40 by transfection with 80 pM of specific siRNAs. Transfection with 80 pM of irrelevant siRNA was used as experimental control. After 48 h, the knock-down of CD40 was verified by qRT-PCR and FACS analysis (data not shown) before using engineered EC for all *in vitro* assays.

**Supplementary Methods S4: *In vitro* studies on Vascular Smooth Muscle Cells (VSMC)**

*Cell calcification:* intracellular calcium deposits were quantified by red alizarin staining. VSMC were cultured in 24-well plates and fixed with 4% paraformaldehyde in PBS for 45 min at 4°C. Cells were then washed in distilled water, exposed to red alizarin (2% aqueous, Sigma Aldrich, St Louis, MO) for 5 min, washed again and analyzed by inverted light microscopy. Cell lysates were analyzed in an automatized spectrophotometer at a wavelength of 570 nm (references 5, 26 main text).

*Quantitative RT-PCR and FACS analysis for Runx2 expression*: total RNA was extracted by VSMC after appropriate stimulation using mirVana RNA isolation kit (Life Technologies). RNA concentration and purity were detected by the NanoDrop1000 spectrophotometer. We evaluated Runx2 mRNA expression by using High cDNA Reverse Transcription Kit (Applied Biosystems, Foster City, CA) and the Power SYBR Green PCR Master Mix on a 96-well StepOnePlus Real Time System (Applied Biosystems, Waltham, MA). Actin-β was used as housekeeping gene. Fold change in RNA expression was calculated as 2-ΔΔCt using the geometric mean in Ct values as normalizer. The following primers were used: • Actin-β: forward, 5’-GAG TCC GGC CCC TCC AT-3’; reverse; 5’-GCA ACT AAG TCA TAG TCC GCC TAG A-3’ • RUNX2: forward 5'- GGA GTG GAC GAG GCA AGA GTT -3'; reverse 5'- CTG TCT GTG CCT TCT GGG TTC -3'. For immunofluorescence studies, VSMC cultured on chamber slides (Thermo Scientific, Waltham, MA) were fixed in ethanol/acetic acid 2:1 and incubated with primary rabbit polyclonal antibodies directed to human Runx2 (Santa Cruz Biotechnology). Slides were washed with PBS and then incubated with Alexa Fluor–conjugated secondary antibodies (Life Technologies) for 30 min at 4°C. All samples were counterstained by 2.5 µg/ml Hoechst (Sigma Aldrich, St. Louis, MO) for 5 minutes, mounted with anti-fade mounting medium (Sigma Aldrich), and examined by confocal microscopy (LSM5 PASCAL; Zeiss, Jena, Germany). For FACS analysis, cells cultured in 12-well plates were detached with EDTA and stained for 30 min at 4°C with primary antibodies directed to human Runx2 (Santa Cruz Biotechnologies). After washing, VSMC were incubated with FITC–conjugated secondary antibodies (Sigma Aldrich) for 30 min at 4°C. All incubation periods were performed using a medium containing 0.25% BSA and 0.0016% sodium azide (Sigma Aldrich). Finally, cells were newly washed, fixed in 4% paraformaldehyde, and analyzed by FACS (Becton Dickinson).

*RNA interference for CD40:* in selected experiments, VSMC were seeded on 24-well plates and engineered to knock-down CD40 by transfection with 80 pM of specific siRNAs. Transfection with 80 pM of irrelevant siRNA was used as experimental control. After 48 h, the knock-down of CD40 was verified by qRT-PCR and FACS analysis (data not shown) before using engineered VSMC for all *in vitro* assays.
